# Injection Molding and Mechanical Properties of Bio-Based Polymer Nanocomposites

**DOI:** 10.3390/ma11040613

**Published:** 2018-04-17

**Authors:** Maria Chiara Mistretta, Luigi Botta, Marco Morreale, Sebastiano Rifici, Manuela Ceraulo, Francesco Paolo La Mantia

**Affiliations:** 1 Department of Civil, Environmental, Aerospace, Materials Engineering, RU INSTM of Palermo, University of Palermo, Viale delle Scienze ed.6, 90128 Palermo, Italy; mariachiara.mistretta@unipa.it (M.C.M.); luigi.botta@unipa.it (L.B.); rifici.sebastiano@gmail.com (S.R.); manuela.ceraulo@unipa.it (M.C.); 2 Faculty of Engineering and Architecture, Kore University of Enna, Viale delle Olimpiadi, 94100 Enna, Italy

**Keywords:** processing, injection molding, biodegradable polymers, nanocomposites

## Abstract

The use of biodegradable/bio-based polymers is of great importance in addressing several issues related to environmental protection, public health, and new, stricter legislation. Yet some applications require improved properties (such as barrier or mechanical properties), suggesting the use of nanosized fillers in order to obtain bio-based polymer nanocomposites. In this work, bionanocomposites based on two different biodegradable polymers (coming from the Bioflex and MaterBi families) and two different nanosized fillers (organo-modified clay and hydrophobic-coated precipitated calcium carbonate) were prepared and compared with traditional nanocomposites with high-density polyethylene (HDPE) as matrix. In particular, the injection molding processability, as well as the mechanical and rheological properties of the so-obtained bionanocomposites were investigated. It was found that the processability of the two biodegradable polymers and the related nanocomposites can be compared to that of the HDPE-based systems and that, in general, the bio-based systems can be taken into account as suitable alternatives.

## 1. Introduction

Several reasons, including issues regarding environmental protection, public health, as well as new legislative rules, are currently encouraging research on the development of biomaterials from renewable sources. The use of biodegradable polymers is particularly promising in terms of reducing the environmental impacts related to the disposal of plastics in landfills, as well as those related to the consumption of nonrenewable resources, since many biodegradable polymers come from renewable resources. Since the packaging industry typically uses large amounts of plastics and often deals with biodegradable products (food, etc.), the use of biodegradable polymers is particularly advisable [1,2].

Among bio-based/biodegradable polymers, poly(lactic acid) (PLA) is probably the most well-known; at the same time, bio-based polymer blends are gaining increasing attention, and are also attracting commercial attention. Among these, Mater-Bi^®^ and Bioflex^®^ can be cited. Mater-Bi is a family of proprietary composition thermoplastic polymers, usually based on starch and/or thermoplastic copolyesters, with multiple, differing synthetic components as well as proportions [3,4], thus covering a relatively wide range of physic-chemical properties. Bioflex is also a family of proprietary composition thermoplastic polymers, but in this case they should be usually based on PLA and biodegradable copolyesters [5].

In general, both PLA-based and MaterBi-based systems can show similar mechanical behaviors to petroleum-based polymers and are compostable, due to their high biodegradation rates and nontoxicity features [3,6].

At the same time, polymer nanocomposites have been receiving huge attention as well, since it is known that even very low amounts of nanosized filler can lead to significant improvements of mechanical, rheological, and barrier properties [2,7,8,9,10,11,12]; in particular, the presence of small amounts of nanosized fillers can lead to physical properties (mechanical, rheological, etc.), which are better, or comparable to those obtained using corresponding microsized fillers [13].

Therefore, it can be of great interest to investigate the properties of bionanocomposites, i.e., nanocomposites based on bioderived/biodegradable thermoplastic matrices in replacement of traditional ones.

PLA-based bionanocomposites are well-reported. PLA is the most investigated biodegradable polymer, partly due to the possibility of improving its mechanical properties.

Ogata et al. [14] prepared PLA/clay composites by blending poly(l-lactide) (PLLA) and organophilic montmorillonite via solvent casting in chloroform. They found an increase of the elastic modulus, due to the intercalation of the silicate lamellae, while delamination (and thus, exfoliation) did not occur.

Chang et al. [15] studied the thermomechanical properties, morphology, and gas permeability of PLA nanocomposites with different organoclays. They prepared (via solvent method) composites with Hexadecylamine–montmorillonite (C16–MMT), dodecyltrimethyl ammonium bromide–montmorillonite (DTA-MMT), and Cloisite 25A, finding that all of the obtained systems had higher tensile strength, reaching a maximum strength at relatively low amounts of filler. The barrier properties also significantly increased. This was explained through TEM analysis, which showed how intercalated and, above all, partially exfoliated structures were obtained.

La Mantia et al. [16] studied the mechanical and rheological properties of a new nanobiocomposite, based on PLA and other biodegradable copolyesters, that was filled with organo-modified montmorillonite. They found that the elastic modulus significantly increased upon adding the clay, while the ductility was reduced accordingly. Furthermore, rheological characterization evidenced a peculiar behavior: at lower shear rates (in a rotational rheometer), the viscosity values shown by the nanocomposite were higher than those of the neat polymer matrix; although, at higher shear rates (obtained in a capillary rheometer), the behaviors were opposite. This was attributed to an orientation attained by clay nanoparticles and polymer macromolecules at higher shear rates, leading to a decrease of flow resistance. They also found [17] that clay nanoparticles slightly increase the thermomechanical degradation rates of PLA, especially when the processing time and temperature are increased.

Several other studies on PLA-based nanocomposites were carried out, showing enhanced mechanical and thermal properties [18,19,20,21,22,23].

Qu et al. [24] prepared nanocomposites of PLA and cellulose nanowhiskers by a solvent casting method. Polyethylene glycol (PEG) was used in order to improve the interfacial adhesion between polymer matrix and nanofiller. A significant increase of tensile strength was found, while the improvement in deformability was only marginal, and thus attributed to a limited interaction between the polymer matrix and the cellulose nanowhiskers. They therefore added PEG, finding a 28% increase of the tensile strength and a 25% increase of the elongation at break, in comparison to neat PLA. Furthermore, the optimum nanofiller content was determined to be 3%, with tensile strength and elongation at break worsening at higher amounts. 

Scaffaro et al. [25] compared several processing methods (single-screw extruder, corotating twin-screw extruder, counter-rotating twin-screw compounder) for preparation of a PLA/hydrotalcite nanobiocomposite. The best overall morphology was obtained by the corotating twin-screw extruder, the worst by using the single-screw extruder. Furthermore, rheological characterization pointed out a reduction of the viscosity, due to degradation phenomena occurring during the nanocomposite processing, while mechanical characterization evidenced an increase in the elastic modulus and a decrease of tensile strength and elongation at break. 

Fukushima et al. [26] investigated the influence of montmorillonite and sepiolite on the thermomechanical properties of PLA and polycaprolactone (PCL). It was found that, in both cases, the nanofillers imparted higher enhancements to PLA, likely due to better interactions than with PCL. 

La Mantia et al. [27] studied the effects of elongational flow on the properties of PLA films filled with organo-modified montmorillonite. The presence of the organo-modified clay lead to an increase of the elastic modulus and the elongation at break, that was higher upon increasing the orientation degree; this is explained by considering higher intercalation and exfoliation degrees. 

Scaffaro et al. [28] prepared bionanocomposites based on PLA, graphene nanoparticles (GnPs) and an antibiotic (cyprofloxacin). The mechanical and rheological properties, as well as the influence of GnPs on the antimicrobial properties, were investigated. It was found that the presence of GnPs increased the elastic modulus and allowed the improvement of the release kinetics of the antibiotic, without negatively affecting the overall antimicrobial properties of the obtained systems. 

Chiu et al. [29] studied the effects on functionalized, single-walled carbon nanotubes (SW-CNTs) on the properties of PLA. Their presence resulted in a significant increase of the dimensional stability, the storage modulus, the glass transition temperature, and a reduction of surface resistivity. Wu et al. [30] also found remarkable improvements of thermal and mechanical properties upon using multi-walled carbon nanotubes (MW-CNTs). 

Comparatively, little information is available on bionanocomposites prepared with biodegradable/biobased polymers other than PLA. 

La Mantia et al. [31] investigated the elongational-flow behavior of a MaterBi biodegradable polymer, reinforced with MW-CNTs. The obtained fibers were hot-and-cold-drawn, and resulted in the viscosity being significantly increased because of the presence of the nanotubes. Upon hot-drawing, rigidity and tensile strength became greater when the draw ratio was increased, and this increment was higher in the nanocomposite samples than in the unfilled MaterBi ones. Upon cold-drawing, the enhancement of the mechanical properties of the nanocomposites was proportionally similar to that of the unfilled systems. Consequently, this explained that the presence of CNTs has a limited influence on the mechanical properties of the fibers since, in the solid state, they show a low mobility which hinders further orientation. 

It is clear from this brief literature review that there are not many systematic studies on bionanocomposites based on polymers other than PLA; in particular, there is little information about the processing of such bionanocomposites. In this work, therefore, we focused on the rheological and mechanical characterization of bionanocomposites, prepared using two different biodegradable polymers, one based on PLA (Bioflex family) and one coming from the MaterBi family. In addition, two different inorganic nanofillers were used, i.e., organo-modified clay and calcium carbonate. Their properties were directly compared to similar nanocomposites with a traditional, oil-derived polymer matrix (high-density polyethylene). Furthermore, the injection molding processability and the properties of the so-obtained samples were investigated.

## 2. Materials and Methods

### 2.1. Materials

The polymer matrices used in this work are reported in Table 1, with the main data reported in the technical datasheets.

Clearflex MP90U is a high-density polyethylene (HDPE) suitable for injection molding operations and used as a reference sample (for comparison purposes). MaterBi has proprietary composition [1,32], but based on available information, it should be probably manufactured using biodegradable copolyesters [31,33]. Bioflex is a biodegradable blend of proprietary composition, generally based on PLA and also containing biodegradable thermoplastic copolyesters and additives [2,34,35].

Nanosized fillers for the preparation of biodegradable nanocomposites were a clay and a calcium carbonate sample, respectively. The nanoclay was a Cloisite 20A (Southern Clay Products, Gonzales, TX, USA) sample, i.e., a ditallowdimethylammonium-modified montmorillonite with an organo-modifier concentration of 95 meq/100 g. The nanosized calcium carbonate was a Socal 312 (Solvay, Bruxelles, Belgium), i.e., a precipitated calcium carbonate with a hydrophobic coating, a rombohedral calcite crystalline structure, and a mean particle diameter of 50–100 nm [13].

In order to protect the two biodegradable polymer matrices from possible hydrolytic scission during processing, they were dried for 4 h under vacuum at 60 °C, while Cloisite (CL20A) was dried for 12 h at 120 °C and Socal 312 (S312) for 12 h at 90 °C. A further exsiccation step (4 h, 60 °C, under vacuum) was performed on all the related specimens, before the following mechanical and rheological tests.

### 2.2. Preparation

The first stage of nanocomposites preparation was carried out in an OMC (Saronno, Italy) corotating twin-screw extruder (L/D = 35). Mechanically mixed polymer pellets and nanosized fillers were poured inside the feeder at 95:5 weight ratio and processed with a 200 rpm screw rotation speed. Each nanocomposite was prepared in the same conditions as the corresponding neat polymer; a summary of temperature profiles is reported in Table 2.

The obtained materials were then pelletized and subjected to injection molding in a Negri Bossi EOS-65 industrial injection molding machine (Cologno Monzese, Italy). The main processing parameters are shown in Table 3.

The specimens for the tensile tests were manufactured by compression molding (CM) in a Carver laboratory press (Wabash, IN, USA). Molding temperature was set at 190 °C, compression time was approx. 4 minutes (under ≈ 5 MPa load). Dog-bone tensile test specimens (L = 65 mm, w = 33 mm, t = 0.6–0.8 mm) were collected by cutting them off the compression-molded sheets. 

Tensile characterization was also performed on the specimens directly coming out from the injection molding (IM) step, with dimensions (105 × 33 × 2) mm.

### 2.3. Characterization

Rheological characterization under non-isothermal elongational flow was performed using a CEAST (Turin, Italy) Rheologic 1000 capillary rheometer, with a capillary having a length-to-diameter (L/D) ratio equal to 40.

Mechanical characterization on the compression-molded samples was performed in tensile mode using a Instron (Norwood, MA, USA) mod. 3365 universal machine, according to ASTM D882 [36], operating at a crosshead speed of 1 mm/min; when the deformation was about 3%, the crosshead speed was increased up to 100 mm/min until final rupture. The reproducibility of the results was adequate (±7%).

Injection-molded samples, on the other hand, were tested in a WANCE (Shenzhen, China) ETM502 apparatus, under similar test parameters. Reproducibility was good (±5%).

Impact tests were carried out on the injection-molded samples in Charpy mode by using a Instron (Norwood, MA, USA) Ceast 9050 universal pendulum at 5 J (with a ±8% reproducibility).

Morphological characterization was performed through SEM analysis, using a FEI (Hillsboro, OR, USA) Quanta 200F ESEM on the fracture surfaces of gold-sputtered samples.

## 3. Results and Discussion

### 3.1. Rheological Characterization

**Figure 1:**
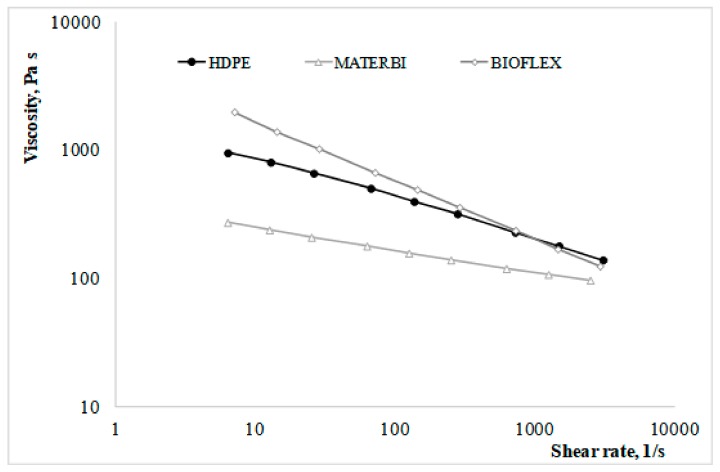
Rheological curves of the three polymers.

Rheological characterization of the prepared systems obviously started from the analysis of the behavior of the two, neat biodegradable polymers and the reference polyolefin. The rheological curves obtained by means of the capillary rheometer are shown in Figure 1.

Although there are significant differences, on average, between the three polymers and especially with regard to the two biodegradable ones (with the MaterBi having much lower viscosities than the Bioflex), the differences at higher shear rates (which are those typical of industrial operations such as injection molding) are remarkably lower, and the overall viscosity values are quite comparable to those of the reference nonbiodegradable polymer. Therefore, in order to assess the actual suitability to injection molding, it became even more important to compare the differences between the three polymers when combined with the same nanofiller (i.e., the different behavior of a nanocomposite upon changing the polymer matrix), with the two different nanofillers when combined with the same polymer matrix (i.e., the role of the nanofiller type in influencing the final properties of the nanocomposite). 

**Figure 2:**
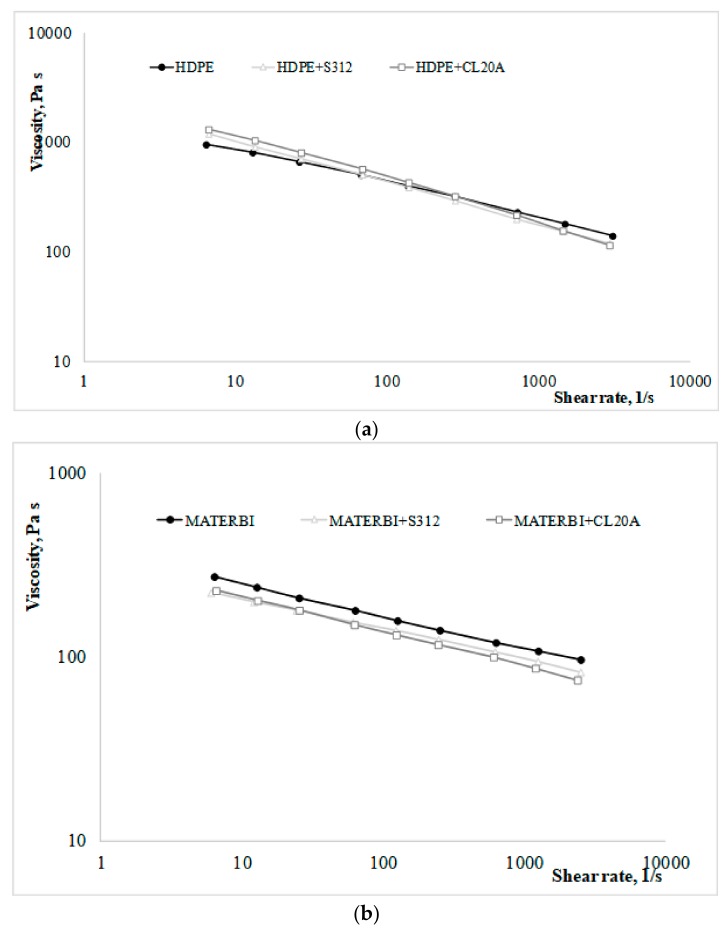

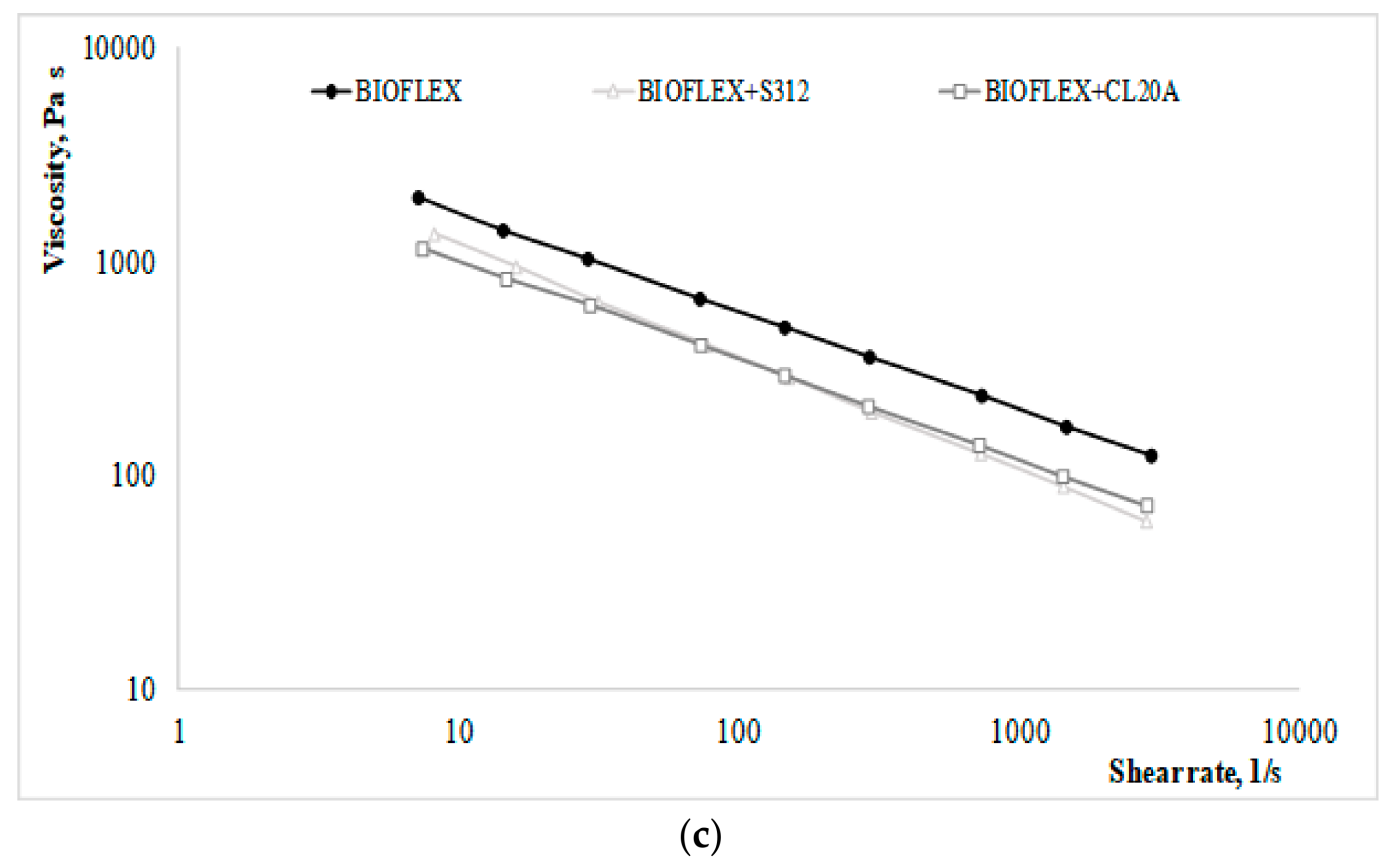
(**a**) Rheological curves of high-density polyethylene (HDPE) without and with a nanofiller; (**b**) rheological curves of MaterBi without and with a nanofiller; (**c**) rheological curves of Bioflex without and with a nanofiller.

The influence of the nanofiller (CL20A or S312) on the overall rheological behavior can be assessed by comparing the rheological curves obtained with the same polymer matrix, as reported in Figure 2a–c.

Concerning the systems with HDPE as matrix (Figure 2a), the presence of CL20A or S312 leads to viscosity values which are very similar to those of the neat matrix, thus assuring an adequate processability (i.e., similar to that of the neat HDPE) of the non-biodegradable nanocomposites. In particular, at higher shear rates (the typical processing range of injection molding operations) the nanocomposites show slightly lower values of viscosity. Therefore, the nanosized fillers display a trend in opposition to that which is typically observed in microcomposites, in agreement with the findings in similar systems [13,37,38].

Similar considerations can be made concerning the MaterBi-based bionanocomposites (Figure 2b). In this case, the gaps between these and the neat matrix are slightly higher. This is even more evident as far as Bioflex-based bionanocomposites are concerned (Figure 2c). The lower viscosity may be due to a degradative effect caused by the nanosized fillers on the biodegradable polymer matrices. In particular, higher differences observed in the Bioflex nanocomposites in comparison to the neat matrix are likely to be due to a degradation effect of the CL20A on the PLA component of the Bioflex matrix [37,39]. Similar considerations apply to the effect S312 may have on the PLA component [38]. In both cases, the systems with CL20A show a more non-Newtonian behavior than those with S312, and this is probably due to the alignment of the silicate lamellae along the flow direction at higher shear rates, that decreases resistance to the flow [37].

**Figure 3:**
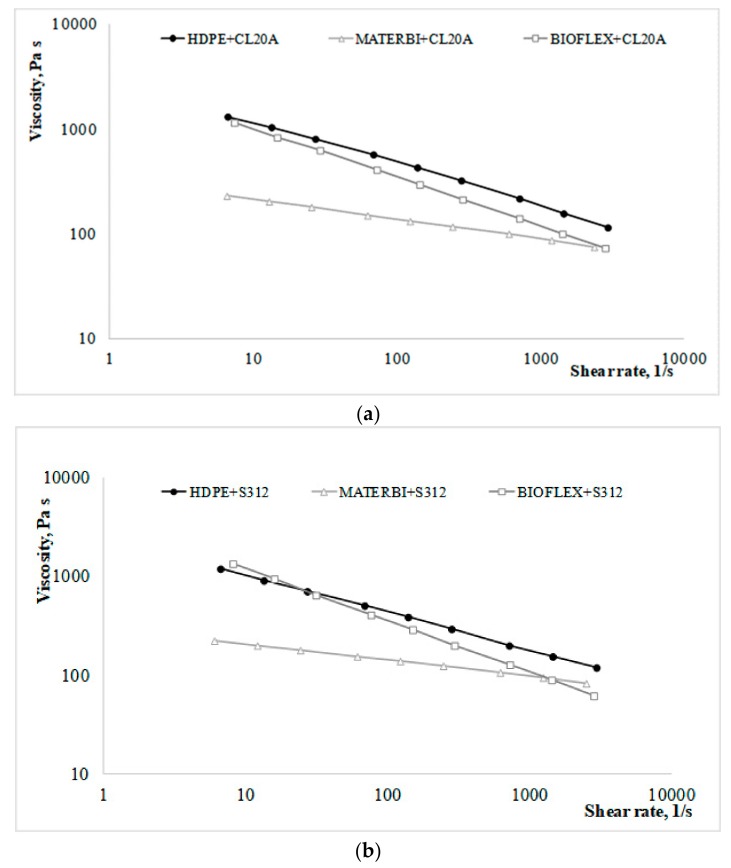
(**a**) Rheological curves of the different CL20A-containing nanocomposites; (**b**) Rheological curves of the different S312-containing nanocomposites.

Concerning the effect of the different polymer matrix on the properties of a nanocomposite prepared with the same nanofiller (either CL20A or S312), it is worth taking into account the rheological curves in Figure 3a,b.

From the reported curves it is clear that, although significant differences occur between the matrices at lower shear rates, the differences at higher shear rates are quite low, and (in particular) negligible as far as the two biodegradable polymers are concerned. In other words, both of the two biodegradable polymers are suitable to injection molding operations in replacement of HDPE.

### 3.2. Mechanical Characterization

**Figure 4:**
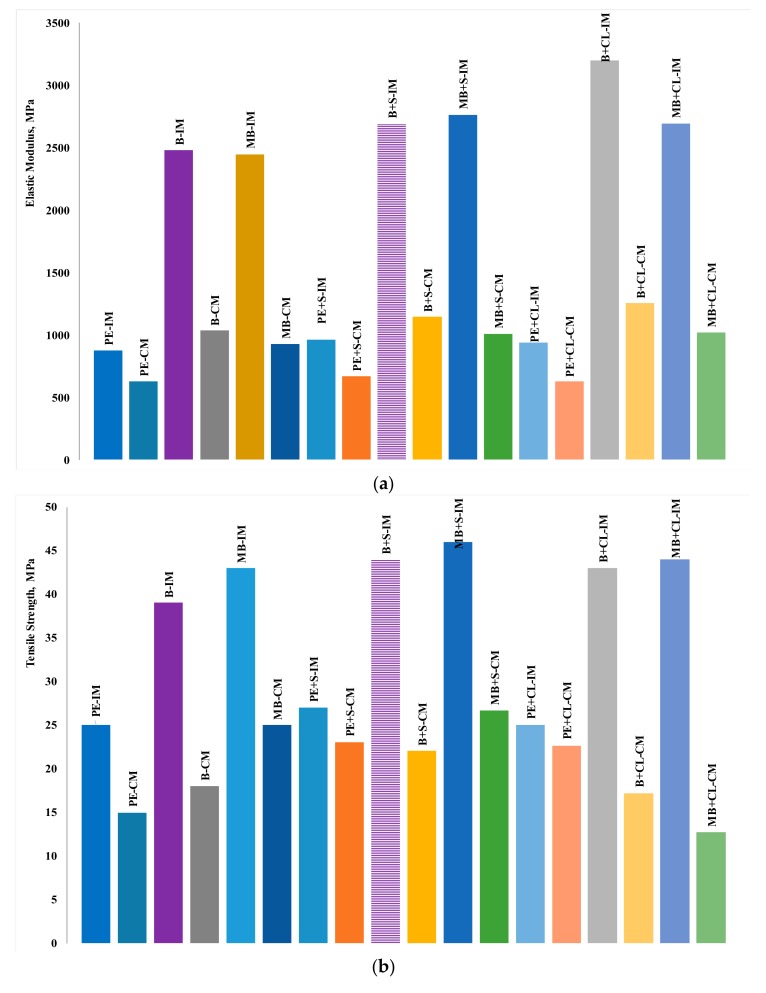

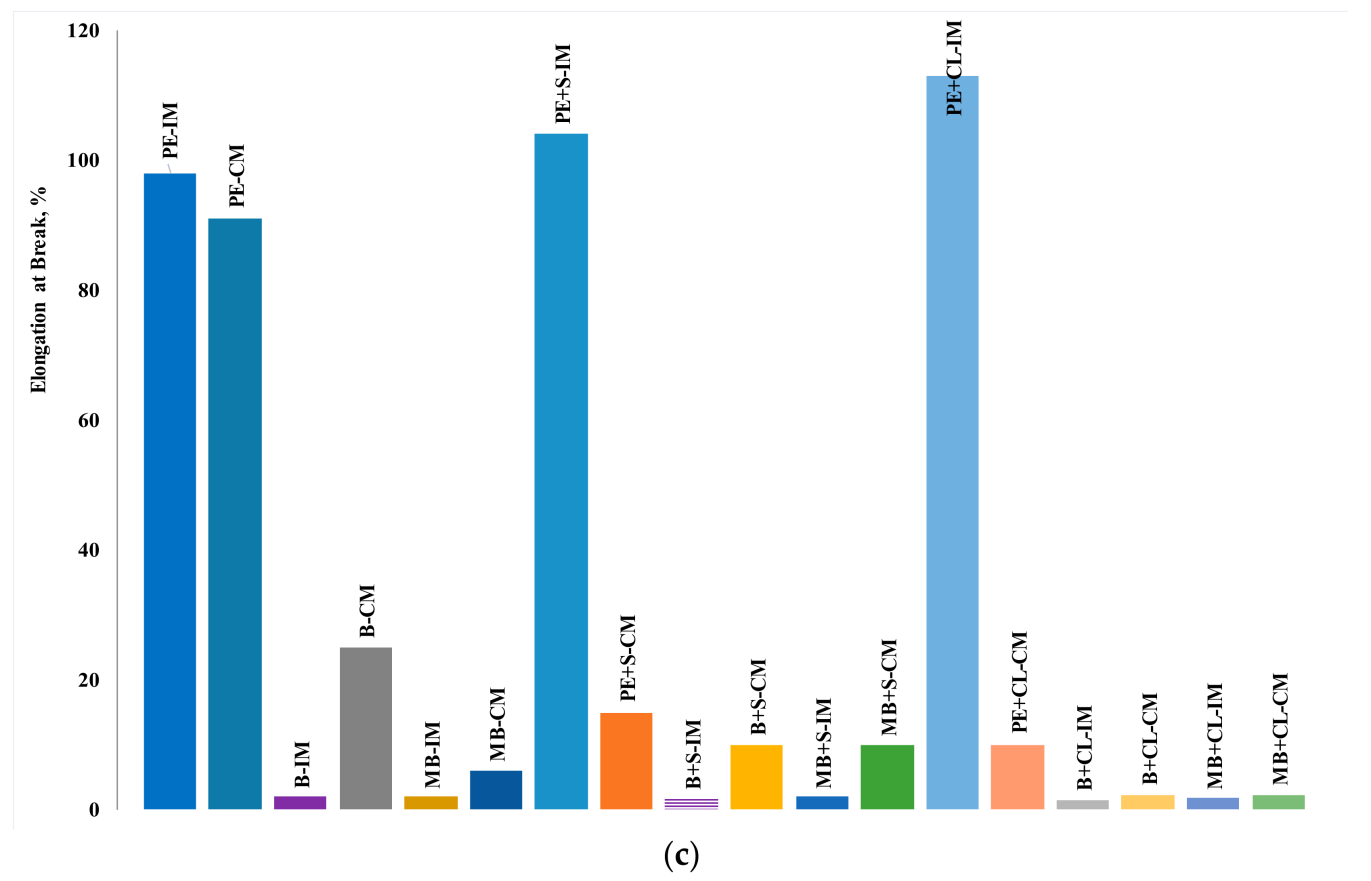
(**a**) Elastic modulus of compression-molded (CM) and injection-molded (IM) samples. (**b**) Tensile strength of compression-molded (CM) and injection-molded (IM) samples. (**c**) Elongation at break of compression-molded (CM) and injection-molded (IM) samples; (PE = HDPE, B = Bioflex, MB = MaterBi, S = calcium carbonate, CL = clay).

The main tensile properties (elastic modulus, E; tensile strength, TS; elongation at break, EB) of compression-molded and injection-molded samples are directly compared. Figure 4a–c, show E, TS and EB, respectively, for the neat polymer (unfilled) samples.

In general, it can be observed that both of the biodegradable polymers show significantly higher values of E and TS, although the deformability is also significantly reduced. Another important factor is the strikingly different mechanical behavior of the injection-molded samples, which is far superior than that of the compression-molded ones, except for the reduction of the ductility. This may be due to an improvement of the material compactness and homogeneity (especially with regard to the homogeneity of the distribution of nanofiller particles in the matrix), achieved during the injection molding process, and can be investigated in more detail by morphological analysis. Furthermore, it is likely that the better performance is related also to reduced thermal degradation effects experienced by the material during the injection molding step, in comparison to the compression molding one; the residence time is significantly higher in the latter, and this can obviously lead to increased thermal stress on the molten material. In particular, thermal degradation can be higher in the case of blends [40], as well as in nanocomposites containing modified clay or modified CaCO_3_ [17,37,38].

Further differences can be discussed between the systems filled with nanosized calcium carbonate (S312). Once more, the properties of the injection-molded samples are better than those of the compression-molded ones. With regard to the effects of the presence of the nanofiller, in comparison to the unfilled counterparts, it can be stated that it leads to an improvement of E and TS in all of the systems, while EB has a slight reduction in the case of the biodegradable samples. 

With regard to effects due to the presence of the other nanofiller, i.e., the organo-modified clay (CL20A), it may be observed that, in comparison to S312, the differences in the obtained effects are small. However, of the two polymers, Bioflex seems to show overall better properties in comparison to MaterBi (although the differences are, as explained before, limited). Furthermore, if compared with our previous studies on injection-molded microcomposites based on MaterBi, it can be observed that lower amounts of filler allowed obtaining, in this case, improvements comparable to those obtained with microsized fillers [4].

In addition to tensile tests, impact tests were performed on the injection-molded systems and the results are shown in Table 4. The impact strength trend reflected the increased brittleness upon adding the nanosized fillers to the neat polymer matrices. Furthermore, impact strength of the two bionanocomposites was lower than that of the HDPE-bases nanocomposites, in agreement with the reduced ductility found from tensile tests.

### 3.3. Morphological Characterization

**Figure 5:**
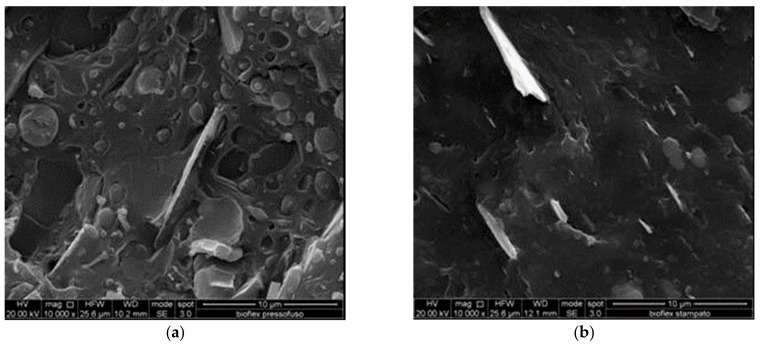
(**a**) SEM fracture surfaces of B-CM sample; (**b**) SEM fracture surfaces of B-IM sample.

In order to obtain a deeper understanding of the different mechanical properties shown by the nanocomposites, upon using different nanofillers and being subjected to different processing, SEM micrographs of the fracture surfaces were taken. Figure 5a,b show the fracture surfaces of compression-molded and injection-molded Bioflex, respectively. It can be easily observed that the morphology of the injection-molded sample is smoother and more homogeneous, probably due to the higher pressures achieved during injection molding, in comparison to compression molding.

**Figure 6:**
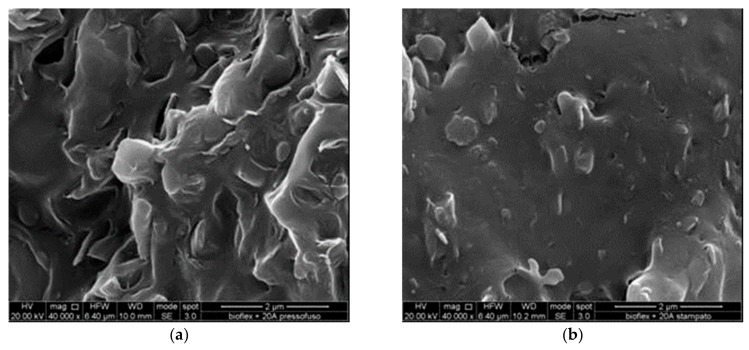
(**a**) SEM fracture surfaces of B+CL-CM sample; (**b**) SEM fracture surfaces of B+CL-IM sample.

Further evidence of this trend was found through observing the fracture surfaces of the nanocomposites. Figure 6a,b show in detail the compression-molded and the injection-molded CL20A nanocomposites, respectively. It can be observed that the injection-molded sample is significantly better in terms of the homogeneity of the polymer matrix (i.e., increased compactness, with lower number of voids and points appearing different from the surrounding ones) and dispersion of the filler (no significant aggregates are visible). The former can be due to the higher pressures achieved, the latter is probably due to the additional extrusion process in the injection molding machine itself. On the basis of our previous works on clay nanocomposites, it can be stated that those visible in Figure 6 are not clay nanoplatelets but tactoids, and that the obtained nanocomposites are intercalated [16,27,32,37].

**Figure 7:**
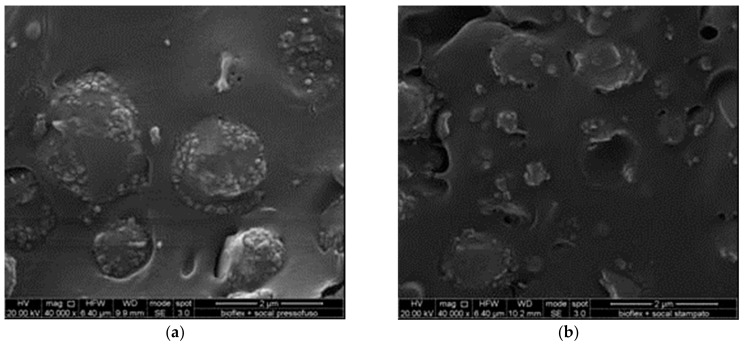
(**a**) SEM fracture surfaces of B+S-CM sample; (**b**) SEM fracture surfaces of B+S-IM sample.

Finally, Figure 7a,b show the compression-molded and the injection-molded S312 nanocomposites, respectively. In the case of the compression-molded sample, large domains of a second phase of the blend are clearly visible (in fact Bioflex is, fundamentally, a blend) and, in particular, the nanofiller particles appear to agglomerate into that phase. The injection-molded sample shows an enhanced homogeneity, i.e., a better dispersion of the second-phase domains and as a consequence (at least partially) of the calcium carbonate nanoparticles. This can account for the better mechanical properties found.

## 4. Conclusions

In this work, bionanocomposites based on two different biodegradable polymers (coming from the Bioflex and MaterBi families) and two different nanosized fillers (organo-modified clay and hydrophobic-coated precipitated calcium carbonate) were prepared and compared with traditional nanocomposites with HDPE as matrix.

Rheological characterization pointed out that the processability of nanocomposites based on Bioflex was relatively close to that of the HDPE-based ones. Tensile characterization of compression-molded samples showed that the biopolymers have higher rigidity and lower deformability in comparison to HDPE, and this trend was moderately emphasized by the presence of the nanofillers. The injection molding processability and the properties of the so-obtained samples were investigated, finding that both of the biopolymers significantly benefit from this processing technique in comparison to compression molding, with enhancements of both the elastic modulus and the tensile strength, and only small reductions of the elongation at break; morphological analysis showed significantly improved nanofiller dispersion and matrix-filler adhesion. Therefore, further mechanical tests (impact) were performed only on the injection-molded samples, confirming that both of the biopolymers show comparable behaviors, although the impact strength clearly reflected the reduced ductility of the bionanocomposites.

The overall results thus demonstrate that both the Bioflex and the MaterBi can be regarded as valid and more environment-friendly alternatives for HDPE in the production of injection-molded items.

## Figures and Tables

**Table 1:** Materials used in this work and related codes.

Material	Manufacturer	MFI, g/10 min	Sample Code
Clearflex MP90U	Versalis (Italy)	7	HDPE
MaterBi D01A	Novamont (Italy)	35	MB
Bioflex F6510	FKuR (Germany)	2.5–4.5	BF

**Table 2:** Processing conditions in the twin-screw extruder.

Sample	Temperature Profile, °C
HDPE + CL20A	120-130-140-150-160-170-190
HDPE + S312	120-130-140-150-160-170-190
BF + CL20A	170-180-180-180-190-190-190
BF + S312	170-180-180-180-190-190-190
MB + CL20A	150-160-170-180-180-190-190
MB + S312	150-160-170-180-180-190-190

**Table 3:** Main injection molding process parameters.

Sample	Temperature Profile, °C	Injection Pressure, Bar	Holding Pressure, Bar
HDPE	235/235/235/240	80	45
HDPE + CL20A	235/235/235/240	80	45
HDPE + S312	235/235/235/240	80	45
BF	165/170/170/170	120	50
BF + CL20A	165/170/170/180	120	50
BF + S312	165/170/170/170	120	50
MB	150/160/170/180	90	50
MB + CL20A	150/160/170/180	90	50
MB + S312	150/160/170/180	90	50

**Table 4:** Impact properties of the injection-molded (IM) systems (NB = no break occurred under these test conditions).

Property	PE-IM	B-IM	MB-IM	PE + S-IM	PE + CL-IM	B + S-IM	B + CL-IM	MB + S-IM	MB + CL-IM
**Impact strength [KJ/m^2^]**	NB	78.18	63.05	NB	NB	20.26	20.92	20.21	23.41

## References

[1] Morreale M., Mistretta M.C., Ceraulo M., La Mantia F.P. (2014). Rheological behavior under shear and non-isothermal elongational flow of biodegradable polymers for foam extrusion. J. Polym. Environ..

[2] Morreale M., Liga A., Mistretta M.C., Ascione L., Mantia F.P.L. (2015). Mechanical, Thermomechanical and Reprocessing Behavior of Green Composites from Biodegradable Polymer and Wood Flour. Materials.

[3] Bastioli C. (1998). Properties and applications of mater-Bi starch-based materials. Polym. Degrad. Stab..

[4] Scaffaro R., Morreale M., Lo Re G., La Mantia F.P. (2009). Effect of the processing techniques on the properties of ecocomposites based on vegetable oil-derived Mater-Bi^®^ and wood flour. J. Appl. Polym. Sci..

[5] Sedlarik V., Saha N., Sedlarikova J., Saha P. (2008). Biodegradation of blown films based on poly(lactic acid) under natural conditions. Macromol. Symp..

[6] Wang L., Tong Z., Ingram L.O., Cheng Q., Matthews S. (2013). Green Composites of Poly(lactic acid) and Sugarcane Bagasse Residues from Bio-refinery Processes. J. Polym. Environ..

[7] Ray S.S., Okamoto K., Okamoto M. (2003). Structure—Property Relationship in Biodegradable Poly(butylene succinate)/Layered Silicate Nanocomposites. Macromolecules.

[8] Giannelis E.P. (1998). Polymer-layered silicate nanocomposites: Synthesis, properties and applications. Appl. Organomet. Chem..

[9] Alexandre M., Dubois P. (2000). Polymer-layered silicate nanocomposites: Preparation, properties and uses of a new class of materials. Mater. Sci. Eng. Rep..

[10] Ray S.S., Okamoto M. (2003). Polymer/layered silicate nanocomposites: A review from preparation to processing. Prog. Polym. Sci..

[11] Choudalakis G., Gotsis A.D. (2009). Permeability of polymer/clay nanocomposites: A review. Eur. Polym. J..

[12] Jancar J., Douglas J.F., Starr F.W., Kumar S.K., Cassagnau P., Lesser A.J., Sternstein S.S., Buehler M.J. (2010). Current issues in research on structure—Property relationships in polymer nanocomposites. Polymer.

[13] La Mantia F.P., Morreale M., Scaffaro R., Tulone S. (2013). Rheological and mechanical behavior of LDPE/calcium carbonate nanocomposites and microcomposites. J. Appl. Polym. Sci..

[14] Ogata N., Jimenez G., Kawai H., Ogihara T. (1997). Structure and thermal/mechanical properties of poly(l-lactide)-clay blend. J. Polym. Sci. B Polym. Phys..

[15] Chang J.-H., An Y.U., Sur G.S. (2003). Poly(lactic acid) nanocomposites with various organoclays. I. Thermomechanical properties, morphology, and gas permeability. J. Polym. Sci. B Polym. Phys..

[16] La Mantia F.P., Mistretta M.C., Palermo S., Ceraulo M. (2015). Morphology, rheology, and mechanical properties of a new nanobiocomposite. J. Appl. Polym. Sci..

[17] La Mantia F.P., Mistretta M.C., Palermo S., Koci E., Ceraulo M. (2016). Thermomechanical degradation of PLA-based nanobiocomposite. Polym. Adv. Technol..

[18] Sinha R.S., Okamoto K., Yamada K., Okamoto M. (2002). Novel porous ceramic material via burning of polylactide/layered silicate nanocomposite. Nano Lett..

[19] Yamada K., Ueda K., Sinha R.S., Okamoto M. (2002). Preparation and properties of polylactide/layered silicate nanocomposites. Kobunshi Robunshu.

[20] Maiti P., Yamada K., Okamoto M., Ueda K., Okamoto K. (2002). New polylactide/layered silicate Nanocomposites: Role of organoclay. Chem. Mater..

[21] Paul M.A., Alexandre M., Degee P., Calberg C., Jerome R., Dubois P. (2003). Exfoliated polylactide/clay nanocomposites by in-situ coordination-insertion polymerization. Macromol. Rapid Commun..

[22] Lee J.H., Park T.G., Park H.S., Lee D.S., Lee Y.K., Yoon S.C., Nam J.D. (2002). Thermal and mechanical characteristics of poly(l-lactic acid) nanocomposite scaffold. Biomaterials.

[23] Chang J., An Y.U., Cho D., Giannelis E.P. (2003). Poly(lactic acid) nanocomposites: Comparison of their properties with montmorillonite and synthetic mica (II). Polymer.

[24] Qu P., Gao Y., Wu G., Zhang L. (2010). Nanocomposites of poly(lactic acid) reinforced with cellulose nanofibrils. BioResources.

[25] Scaffaro R., Botta L., Passaglia E., Oberhauser W., Frediani M., Di Landro L. (2014). Comparison of Different Processing Methods to Prepare Poly(lactid acid)–Hydrotalcite Composites. Polym. Eng. Sci..

[26] Fukushima K., Tabuani D., Camino G. (2009). Nanocomposites of PLA and PCL based on montmorillonite and sepiolite. Mater. Sci. Eng. C.

[27] La Mantia F.P., Ceraulo M., Mistretta M.C., Sutera F., Ascione L., Nasillo G. (2016). Effect of Elongational Flow and Polarity of Organomodified Clay on Morphology and Mechanical Properties of a PLA Based Nanobiocomposite. Int. Polym. Proc..

[28] Scaffaro R., Botta L., Maio A., Mistretta M.C., La Mantia F.P. (2016). Effect of Graphene Nanoplatelets on the Physical and Antimicrobial Properties of Biopolymer-Based Nanocomposites. Materials.

[29] Chiu W.M., Kuo H.Y. (2013). Preparation and Properties of Poly(Lactic Acid) Nanocomposites Filled with Functionalized Single-Walled Carbon Nanotubes. J. Polym. Environ..

[30] Wu C.S., Liao H.T. (2007). Study on the preparation and characterization of biodegradable polylactide/multi-walled carbon nanotubes nanocomposites. Polymer.

[31] La Mantia F.P., Mistretta M.C., Scaffaro R., Botta L., Ceraulo M. (2015). Processing and characterization of highly oriented fibres of biodegradable nanocomposites. Compos. Part B Eng..

[32] La Mantia F.P., Arrigo R., Morreale M. (2014). Effect of the orientation and rheological behaviour of biodegradable polymer nanocomposites. Eur. Polym. J..

[33] La Mantia F.P., Ceraulo M., Mistretta M.C., Morreale M. (2017). Effect of Cold Drawing on Mechanical Properties of Biodegradable Fibers. J. Appl. Biomater. Funct. Mater..

[34] FKuR Website. https://fkur.com/en/brands/bio-flex-3/bio-flex-f-2110.

[35] Gregorova A., Riedl E., Sedlarik V., Stelzer F. (2012). Effect of 4,4′-methylenediphenyl diisocyanate on thermal and mechanical properties of Bioflex/lactic acid polycondensate blends. Asia-Pac. J. Chem. Eng..

[36] (2012). Standard Test Method for Tensile Properties of Thin Plastic Sheeting.

[37] La Mantia F.P., Mistretta M.C., Morreale M. (2014). Recycling and Thermomechanical Degradation of LDPE/Modified Clay Nanocomposites. Macromol. Mater. Eng..

[38] Nekhamanurak B., Patanathabutr P., Hongsriphan N. (2014). The Influence of Micro-/Nano-CaCO_3_ on Thermal Stability and Melt Rheology Behavior of Poly(lactic acid). Energy Procedia.

[39] Scaffaro R., Sutera F., Mistretta M.C., Botta L., La Mantia F.P. (2017). Structure-properties relationships in melt reprocessed PLA/hydrotalcites nanocomposites. Express Polym. Lett..

[40] La Mantia F.P., Morreale M., Botta L., Mistretta M.C., Ceraulo M., Scaffaro R. (2017). Degradation of polymer blends: A brief review. Polym. Degrad. Stab..

